# Morphine suppresses the immune function of lung cancer by up-regulating MAEL expression

**DOI:** 10.1186/s40360-022-00632-z

**Published:** 2022-12-07

**Authors:** Qichao Wang, Zhenfu Liu, Shuhong Tang, Zhen Wu

**Affiliations:** 1 Department of Oncology II, Dalian Fifth People’s Hospital, No. 890, Huanghe Road, Shahekou District, Dalian City, 116021 Liaoning Province China; 2 Department of Anesthesiology, Zaozhuang Hospital of Zaozhuang Mining Group, No. 188, Shengli Road, Zaozhuang City, 277100 Shandong Province China; 3 Department of Anesthesiology, The Fourth Hospital of Hebei Medical University, No.12, Jiankang Road, Shijiazhuang City, 050000 Hebei Province China

**Keywords:** Morphine, Immune, MAEL, Lung cancer

## Abstract

**Background:**

Patients with cancer rely on morphine for analgesia, while studies have indicated morphine can induce immunosuppression in cancer. Therefore, investigating the immunosuppressive roles and molecular mechanism of morphine on lung cancer progression is imperative.

**Methods:**

Lactate dehydrogenase (LDH) release assay was used to determine the cytotoxicity of morphine to lung cancer cells. The percentage of CD4^+^ and CD8^+^ T cells was detected by flow cytometry. In addition, Maelstrom (MAEL), Nrf2, and PTEN were determined by western blot and RT-qPCR. Immune factors programmed death-ligand 1 (PD-L1), transforming growth factor (TGF-β), interleukin (IL)-10, and IL-2 were determined by western blot and ELISA assay.

**Results:**

Morphine increased the levels of PD-L1, TGF-β, and IL-10, while decreased IL-2 level. Morphine enhanced MAEL expression in A549 cells and H460 cells. Morphine up-regulated Nrf2 and down-regulated PTEN, and morphine-induced MAEL up-regulation was reversed by PTEN. However, MAEL silencing inhibited the enhanced effects of morphine on cell viability and proliferation of A549 cells. Furthermore, morphine treatment reduced the LDH release and the percentage of CD8^+^ T cells, and increased the ratio of CD4^+^/CD8^+^ T cells and tumor weight. Meanwhile, MAEL silencing reversed the effects of morphine on immune factors (PD-L1, TGF-β, IL-10, and IL-2), the percentage of CD8^+^ T cells, and the ratio of CD4^+^/CD8^+^ T cells.

**Conclusion:**

Morphine activated MAEL in lung cancer cells by Nrf2/PTEN pathway and regulated the immune factors, thereby promoting tumor immune escape.

**Supplementary Information:**

The online version contains supplementary material available at 10.1186/s40360-022-00632-z.

## Background

Lung cancer is a high mortality heterogeneous disease [1]. Approximately 80-85% of human lung cancers are non-small cell lung cancer (NSCLC), and the remaining 15-20% are small cell lung cancer (SCLC) [2]. Lung cancer patients are caused by smoking, while non-smokers are caused by exposure to secondhand smoke [3]. Surgery is the main treatment for patients with early lung cancer, other common treatments for lung cancer patients include radical radiotherapy, radiofrequency/microwave ablation, systemic therapies, immunotherapy, and targeted therapy [4, 5]. Pain is a very common symptom in all stages of cancer patients. Pain suppresses immunity, and immunity is important to prevent cancer [6]. Therefore, it is very important to control cancer-related pain and improve immune function in cancer patients.

Maelstrom (MAEL) is a cancer-testis (cancer-germline) gene, which is principally expressed in germline cells. Furthermore, MAEL is over-expressed in various cancer types including lung, breast, prostate, and colon cancer [7]. MAEL interacts with stress granule proteins and may be part of the ribonucleoprotein complex in breast cancer and colorectal cancer [8]. MAEL up-regulates IL8 through Akt1/RelA to promote esophageal squamous cell carcinoma progression [9]. MAEL exerts its oncogenic function by promoting ILKAP degradation in gastric cancer [10]. Moreover, evidence has suggested that many cancer germline genes are involved in cell proliferation, and their high expression triggers the attack of immune cells in cancer [11]. MAEL protein has two domains, the MAEL-specific domain (MSD) with a putative RNAse-H-like fold and the amino-terminal high mobility group (HMG)-box domain [12, 13], which plays an important role in the innate immune response to immunogenic foreign nucleic acids [14, 15]. However, the role of MAEL on immune regulation in lung cancer is unclear.

Morphine is an opioid widely used in the treatment of pain, but the individual response of patients varies greatly, and there is no clear guidance on the morphine dosage regimen, which complicates clinical treatment [16]. In addition, morphine plays a vital role in the regulation of cancer [17]. It can regulate and suppress the immune system through the central mediated mechanism [18]. Intravenous morphine suppresses the immune and stress of patients with modified radical mastectomy [19]. Morphine increases IL-6 concentration and suppresses natural killer cell activity, thereby suppressing immune response during surgery [20]. Morphine also increases the ratio of CD4^+^/CD8^+^ T cells, thereby inhibiting the immune function of patients with gastric cancer [21]. Moreover, morphine can enhance the growth and spread of some cancer diagnoses, such as NSCLC [18]. Overall survival (OS) is significantly shorter in NSCLC patients taking opioids than in those who didn’t [22]. However, the molecular mechanism of morphine-mediated immunity in lung cancer remains unclear.

In this research, we demonstrated morphine may suppress immune response via promoting MAEL expression in lung cancer cells, and MAEL may be a potential target for clinical research.

## Materials and methods

### Cell culture

The human lung cancer cell lines (A549 and H460 cells) and normal cells (BEAS-2B) were obtained from American Type Culture Collection (Manassas, VA, USA), and cultured in RPMI Medium 1640 (Gibco, Waltham, MA, USA) supplemented with 10% FBS, 1% streptomycin / penicillin at 37 °C in 5% CO_2_ and 95% humidity. A549 cells were randomly divided into control and morphine (treatment with 1, 10, and 20 μM morphine, respectively) groups.

### Transfection

Small interfering RNA targeting MAEL (si-MAEL) and the negative control (si-NC) were obtained from GenePharma (Shanghai, China). Cells were seeded in 24-well plates at 37 °C for 24 h and were transfected with si-MAEL and si-NC for 48 h using Lipofectamine 3000 reagent (Invitrogen, Carlsbad, CA, USA) according to the instructions of manufacturer. Subsequently, the transfected cells were collected for the next experiments.

### Enzyme-linked immunosorbent assay (ELISA)

The levels of programmed cell death ligand 1 (PD-L1), transforming growth factor-beta (TGF-β), interleukin (IL)-10, and IL-2 in the cellular supernatant were detected by ELISA kits (Esebio, Shanghai, China) following the instructions of the manufacturer.

### Western blot assay

Protein was extracted using Protein Extraction Kit (Sigma-Aldrich, St. Louis, MO, USA). The samples were resolved by 10% SDS-PAGE and transferred to the PVDF membrane. Next, the membranes were blocked with 5% skim milk at 25 °C for 1 h, then incubated with the primary antibodies against MAEL (ab106713, 1:100, Abcam, Cambridge, MA, USA), PD-L1 (ab205921, 1:100, Abcam), TGF-β (ab215715, 1:1000, Abcam), IL-2 (ab92381, 1/1000, Abcam), IL-10 (ab133575, 1:1000, Abcam) and GAPDH (ab8245, 1:1000, Abcam) overnight at 4 °C. After, the membranes were incubated with horseradish peroxidase (HRP)-conjugated secondary antibody (1:5000) at 25 °C for 1 h. Blots were analyzed by enhanced chemiluminescence western blotting detection kits (Sigma-Aldrich). GAPDH was employed as a protein loading control.

### Quantitative real-time PCR (qRT-PCR)

The total RNA was extracted using TRIzol reagent (Sigma-Aldrich). RNA was reversed transcribed into cDNA using M-MLV Reverse Transcriptase kit (Sigma-Aldrich). The qRT-PCR was performed using a SYBR® Green PCR Kit (Sigma-Aldrich) according to the instructions of manufacturer. The Mastercycler ep realplex detection system (Eppendorf, Hamburg, Germany) was used for RT-qPCR assay. β-Actin was used for normalization of the mRNA expression. The primer sequences are shown in Table 1.

**Table 1:** Primers for qRT-PCR in this study

Gene	Forward	Reverse
MAEL	5′-TTCCACGAGGATTTCGATTC-3′	5′-TCCATACGCTTCAAACACCA-3′
Nrf2	5′-ACACGGTCCACAGCTCATC-3′	5′-TGCCTCCAAGTATGTCAATA-3′
PTEN	5′-TTGATTGCATCTCCATCTCCT-3′	5′-TTCGCTTTCTCTGAGCATTCT-3′
β-Actin	5′-AAGTGTGACGTTGACATCCG-3′	5′-GATCCACATCTGCTGGAAGG-3′

### Bioinformatics

Gene Expression Profiling Interactive Analysis (GEPIA) (http://gepia.cancer-pku.cn/) was used to analyze the expression of MAEL in lung adenocarcinoma (LUAD).

### 3-[4,5-dimethyl-2-thiazolyl]-2,5 diphenyl-2H-tetrazolium bromide (MTT) assay

The viability of A549 cells was measured using the MTT kit (Sigma-Aldrich). Cells were cultured into 96-well plates for 24 h. Then, 20 μl of MTT (2.5 mg/ml) was added to the wells and maintained at 37 °C for 4-6 h. Afterward, the formazan crystals were dissolved by dimethyl sulfoxide (DMSO) after the medium was removed. Finally, the absorbance was measured at 450 nm, and this was recorded at 0, 24, 48, and 72 h.

### Cell proliferation assay

According to the instructions of manufacturer, the proliferation of A549 cells was detected by the 5-Ethynyl-20-deoxyuridine (EdU) kit (Ribobio, Guangzhou, China). The cell nuclei were counter stained with DAPI (1 mg/ml) for 5 min. Finally, the images were acquired by the fluorescence microscope (Leica, Wetzlar, Germany), and the EdU positive cell ratio was calculated.

### Animals

Male Sprague-Dawley rats (180 ± 20 g, EseBio, Shanghai, China) were used in this experiment. The tumor model was established by subcutaneously injecting 2 × 10^7^ cells in 200 μL PBS into the bilateral flank of the rats.To evaluate the effect of morphine on immune function, the rats were randomly divided into control (*n* = 6) and morphine (*n* = 6, intraperitoneally injected, 0.1, 0.3, and 0.5 mg/kg, respectively) groups. In addition, to explore the interaction between MAEL and morphine on immune function in lung cancer, the rat models were divided into control, morphine, morphine+si-NC, and morphine+si-MAEL groups (n = 6 each group). For the rats of morphine+si-NC and morphine+si-MAEL groups, a total of 0.2 mL of PBS containing A549 cells (2 × 10^7^ cells) with si-NC and si-MAEL were subcutaneously injected into the bilateral flank of the rats. In addition, rats were injected intraperitoneally with morphine (0.3 mg/kg) once daily for 3 days. Rats in the control group were injected with 0.9% saline. At the end of the experiment, the rats were euthanized with pentobarbital sodium. The animal research procedures were carried out in accordance with the protocol approved by the Institutional Animal Care and Use Committee and approved by the committee of Dalian Fifth People’s Hospital.

### Lactate dehydrogenase (LDH) assay

Collect the spleen under aseptic conditions after rats are sacrificed. The spleen sterile Tris-NH_4_Cl is used to remove red blood cells and RPMI-1640 medium was used to make spleen cell suspension (1 × 10^7^ cells/ml). A549 cells (4 × 10^4^) and 8 × 10^5^ spleen cells were added to each well of the 96-well plate to make an effective target ratio of 20:1. Effector cells natural release control, target cells natural release control, target cells max release control, culture medium control, and cell culture medium correction control were set. Each sample was repeated 3 times. After incubation for 4 h, cell lysate (10 μl) was added to the target cells, Max release control wells and culture medium control wells. After incubating for 1 h, 50 μl supernatant was collected from each well and transferred to another 96-well plate. Then, 50 μl LDH substrate was added to each well and incubated at 25 °C for 30 min (avoid light). Stop solution (50 μl) was added and the absorbance (490 nm) was determined via a microplate reader (Bio-Rad, Hercules, CA).

### Flow cytometry

Rats were sacrificed by cervical dislocation. Peripheral blood mononuclear cells (PBMCs) were isolated from fresh heparinized blood samples of rats by density gradient separation using Ficoll density gradient (TBD Science, China). PBMCs were then stained with the PE-anti-CD8, FITC-anti-CD4 (BioLegend, San Diego, USA), and incubated at 4 °C for 30 min (avoid light). Subsequently, cells were washed twice with PBS and re-suspended in a 4% paraformaldehyde solution with a final volume of 300 μL for analysis. The percentage of CD4^+^ T cells and CD8^+^ T cells in peripheral blood were detected by flow cytometry and analyzed by FlowJo VX (Treestar, USA).

### Statistical analysis

Two-group comparisons were analyzed by Student’s t-test. Differences among groups were analyzed by one-way ANOVA with Tukey’s post hoc test and two-way ANOVA by post-hoc t tests corrected with false discovery rate using Prism 5.0 software (GraphPad, La Jolla, CA). Statistical data were presented as the mean ± SD. *P* < 0.05 was considered significant.

## Results

### Morphine regulates immune factors expression and reduces LDH release

**Fig. 1:**
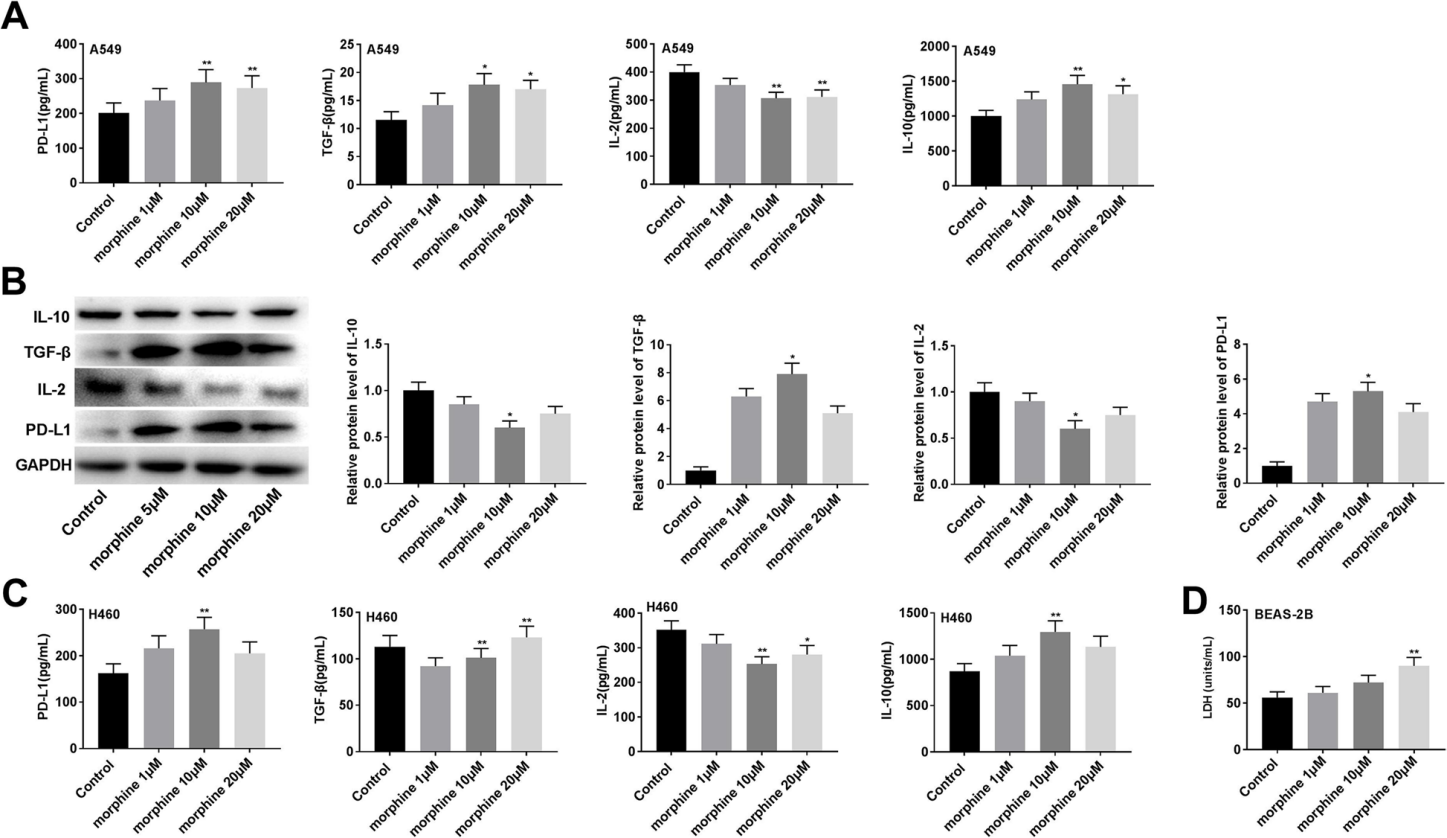
Morphine regulates immune factor expression and reduces LDH release. **A** The expression of PD-L1, TGF-β, IL-2, and IL-10 was detected by ELISA assay in A549 cells. **B** The expression of PD-L1, TGF-β, IL-2, and IL-10 was detected by western blot. **C** The expression of PD-L1, TGF-β, IL-2, and IL-10 was detected by ELISA assay in H460 cells. **D** The LDH release was detected using the LDH kit.*P < 0.05, **P < 0.01 versus control

PD-L1 is highly expressed in various types of cancer and contributes to promoting the tumor immune escape [23]. The activation of TGF-β and IL-10 play an immunosuppressive role in the tumor microenvironment, while IL-2 can enhance the immune response of cancer patients [24–26]. The effects of morphine on immune factors were determined in A549 cells. The result suggested that the expression of PD-L1, TGF-β, and IL-10 were increased in a dose-dependent manner of morphine, while morphine reduced IL-2 expression in a dose-dependent manner (Fig. 1A, *P* < 0.01). Furthermore, these results were further verified by the western blot analysis (Fig. 1B, *P* < 0.01). Meanwhile, similar results are shown in H460 cells, morphine increased the levels of PD-L1, TGF-β, and IL-10, while decreasing the level of IL-2 (Fig. 1C, *P* < 0.05). The results showed that morphine treatment may promote the tumor immune escape by regulating immune factors expression in lung cancer cells. In addition, LDH release assay was used to determine the cytotoxicity of morphine to normal cells. Low concentration morphine treatment had no significant effect on the cytotoxicity of BEAS-2B cells, while 20 μM morphine has toxic to BEAS-2B cells (Fig. 1D, *P* < 0.01).

### Morphine up-regulates MAEL expression in A549 and H460 cells

**Fig. 2:**
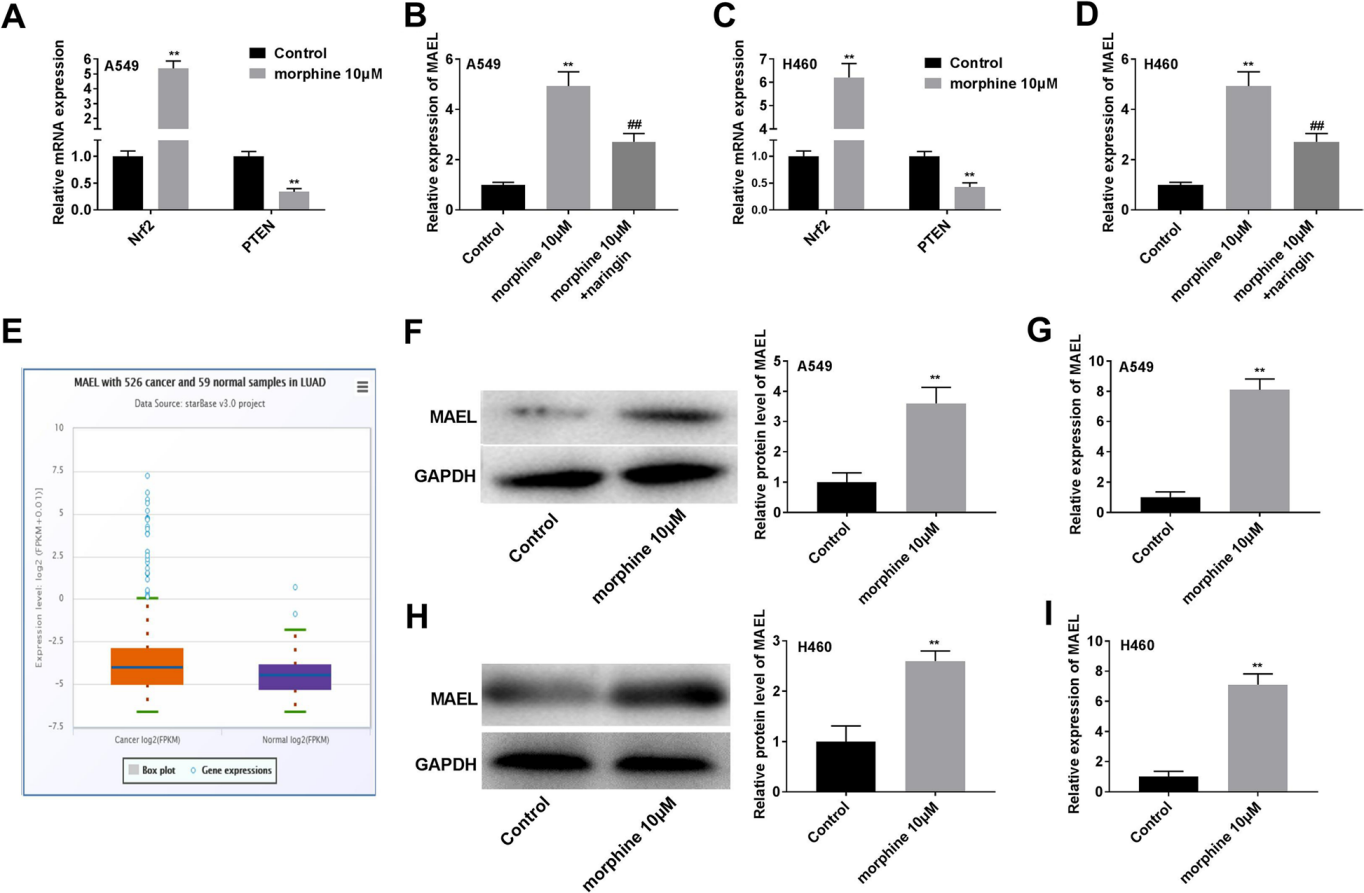
Morphine up-regulates MAEL expression in A549 and H460 cells. **A**-**D** The expression of Nrf2, PTEN, and MAEL was determined in by qRT-PCR. **P < 0.01 versus control. ^##^*P* < 0.01 versus morphine. **E** MAEL expression was determined in LUAD and normal samples. **F**, **G** MAEL expression in A549 cells. **H**, **I** MAEL expression in H460 cells.*P < 0.05, **P < 0.01 versus control

Nuclear factor erythroid 2-related factor 2 (Nrf2) / phosphatase and tensin homologue (PTEN) plays an important role in antitumor immunity [27, 28]. We found morphine increased Nrf2 and decreased PTEN expression in A549 and H460 cells, while naringin (PTEN activator, 150 μM) decreased MAEL expression induced by morphine (Fig. 2A-D, *P* < 0.01), suggesting morphine may regulate MAEL expression by Nrf2/PTEN pathway. The expression of MAEL was determined in lung adenocarcinoma (LUAD) based on GEPIA. As shown in Fig. 2E, MAEL was highly expressed in LUAD compared with the normal samples, while the difference was not significant. Furthermore, morphine treatment up-regulated MAEL expression in A549 and H460 cells (Fig. 2F-I, *P* < 0.01).

### MAEL regulates immune cytokines expression in A549 and H460 cells

**Fig. 3:**
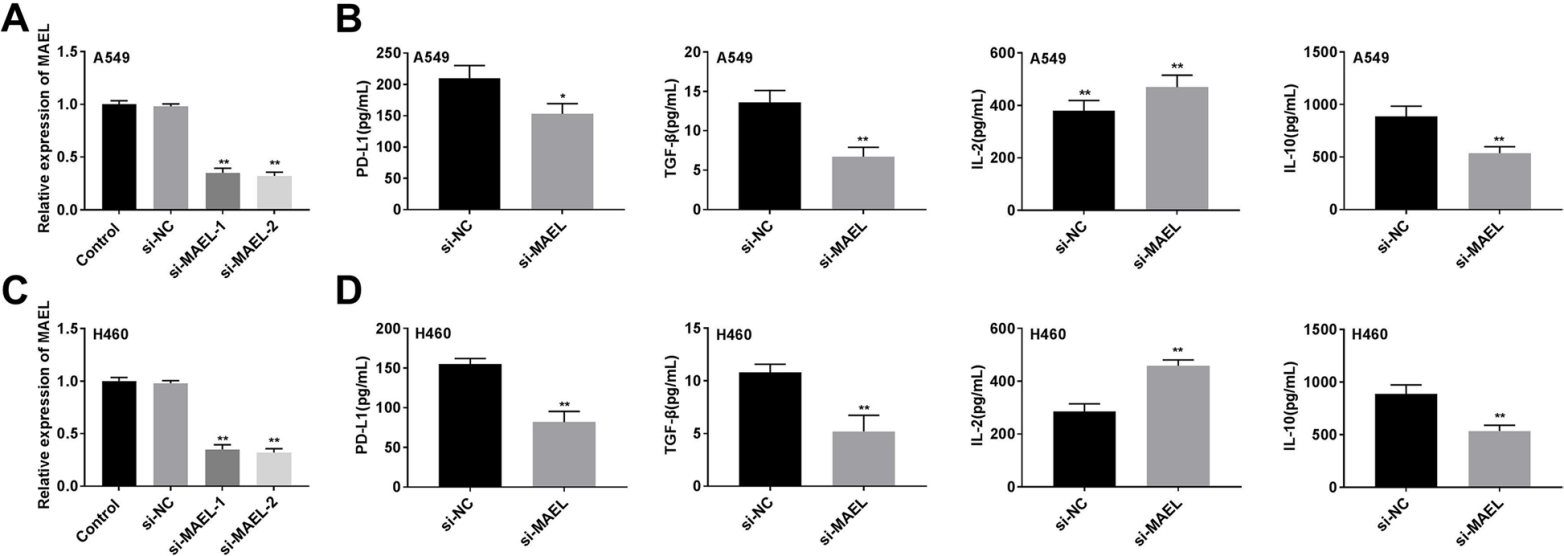
MAEL regulates immune cytokines expression in A549 and H460 cells. **A** MAEL expression was detected by RT-qPCR. **B** The expression of PD-L1, TGF-β, IL-2, and IL-10 in A549 cells. **C** MAEL expression was detected by RT-qPCR in H460 cells. **D** The expression of PD-L1, TGF-β, IL-2, and IL-10 in H460 cells. *P < 0.05, **P < 0.01 versus si-NC

To explore the function of MAEL in lung cancer, MAEL was knocked out by siRNA transfection. The data showed that MAEL expression was decreased after MAEL silencing in A549 cells (Fig. 3A, *P* < 0.01). As shown in Fig. 3, the silencing of MAEL decreased the expression of PD-L1 (*P* < 0.05), TGF-β (*P* < 0.01), and IL-10 (*P* < 0.01), as well as increased the expression of IL-2 (Fig. 3B, *P* < 0.01). In addition, MAEL expression was significantly reduced in H460 cells after transfection with si-MAEL (Fig. 3C, *P* < 0.01). Similarly, MAEL silencing reduced the expression of PD-L1, TGF-β, and IL-10, while increasing the expression of IL-2 in H460 cells (Fig. 3D, *P* < 0.01). The result showed that MAEL may regulate the secretion of immune cytokines in lung cancer cells.

### Morphine promotes proliferation and regulates immune factors by increasing MAEL expression

**Fig. 4:**
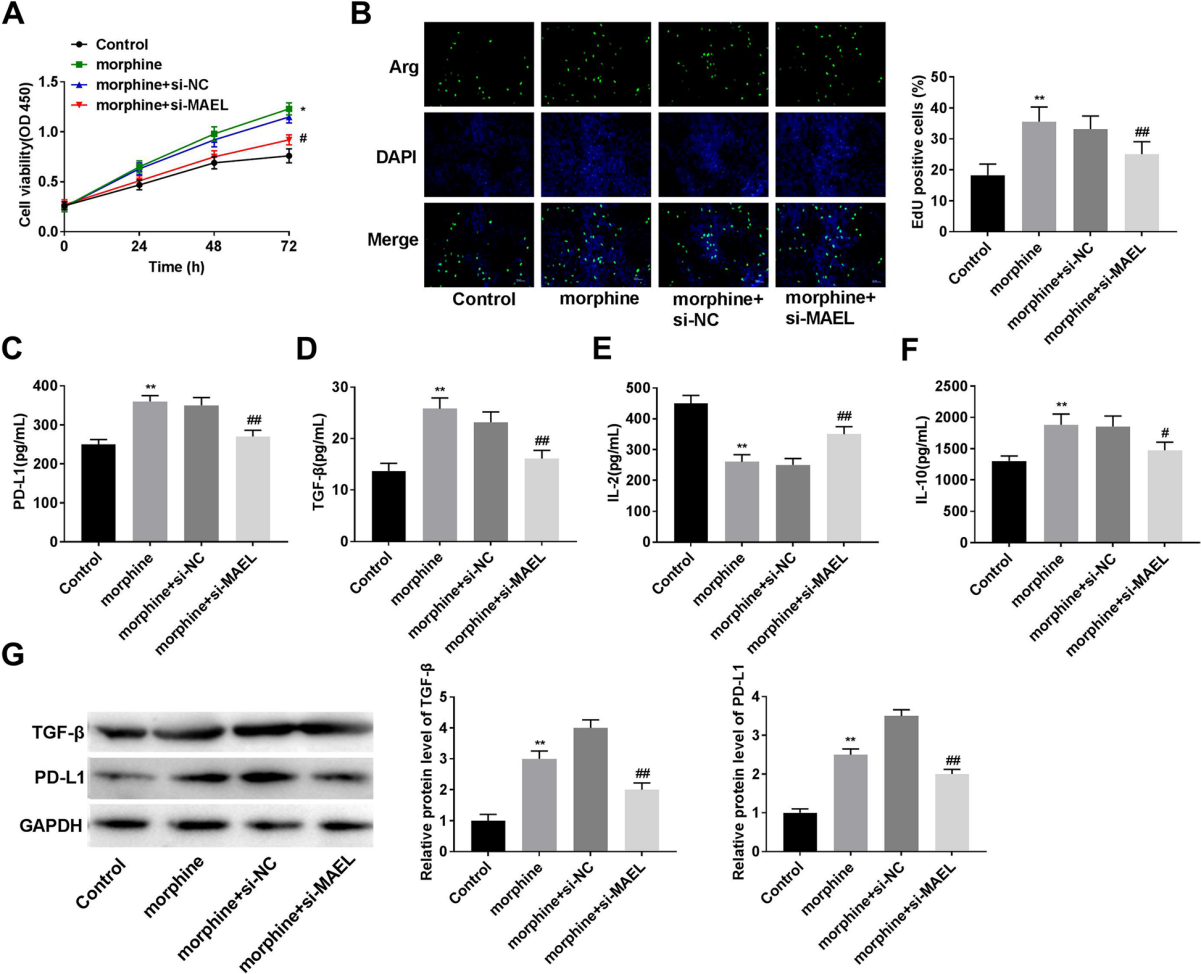
Morphine promotes proliferation and regulates immune factors by increasing MAEL expression. **A** Cell viability was detected by MTT assay. **B** Cell proliferation was detected by the EdU kit. **C**-**F** The expression of PD-L1, TGF-β, IL-2, and IL-10 were detected by ELISA assay. **G** The expression of PD-L1 and TGF-β were detected by western blot. *P < 0.05, **P < 0.01 versus control. ^##^*P* < 0.01 versus morphine+si-NC

Morphine treatment increased cell viability, while MAEL silencing inhibited the increase of cell viability by morphine (Fig. 4A, *P* < 0.05). Meanwhile, morphine treatment increased EdU positive cells, while MAEL silencing inhibited the increase of EdU positive cells by morphine (Fig. 4B, *P* < 0.01). Furthermore, MAEL silencing reversed the effects of morphine on PD-L1, TGF-β, IL-10, and IL-2 (Fig. 4C-G, *P* < 0.01). The data showed that silencing of MAEL attenuates the effects of morphine on cell viability, proliferation, and immune factors.

### Morphine decreases LDH release and the percentage of CD8^+^ T cells, increased the ratio of CD4^+^/CD8^+^ T cells, and promoted the tumor weight

**Fig. 5:**
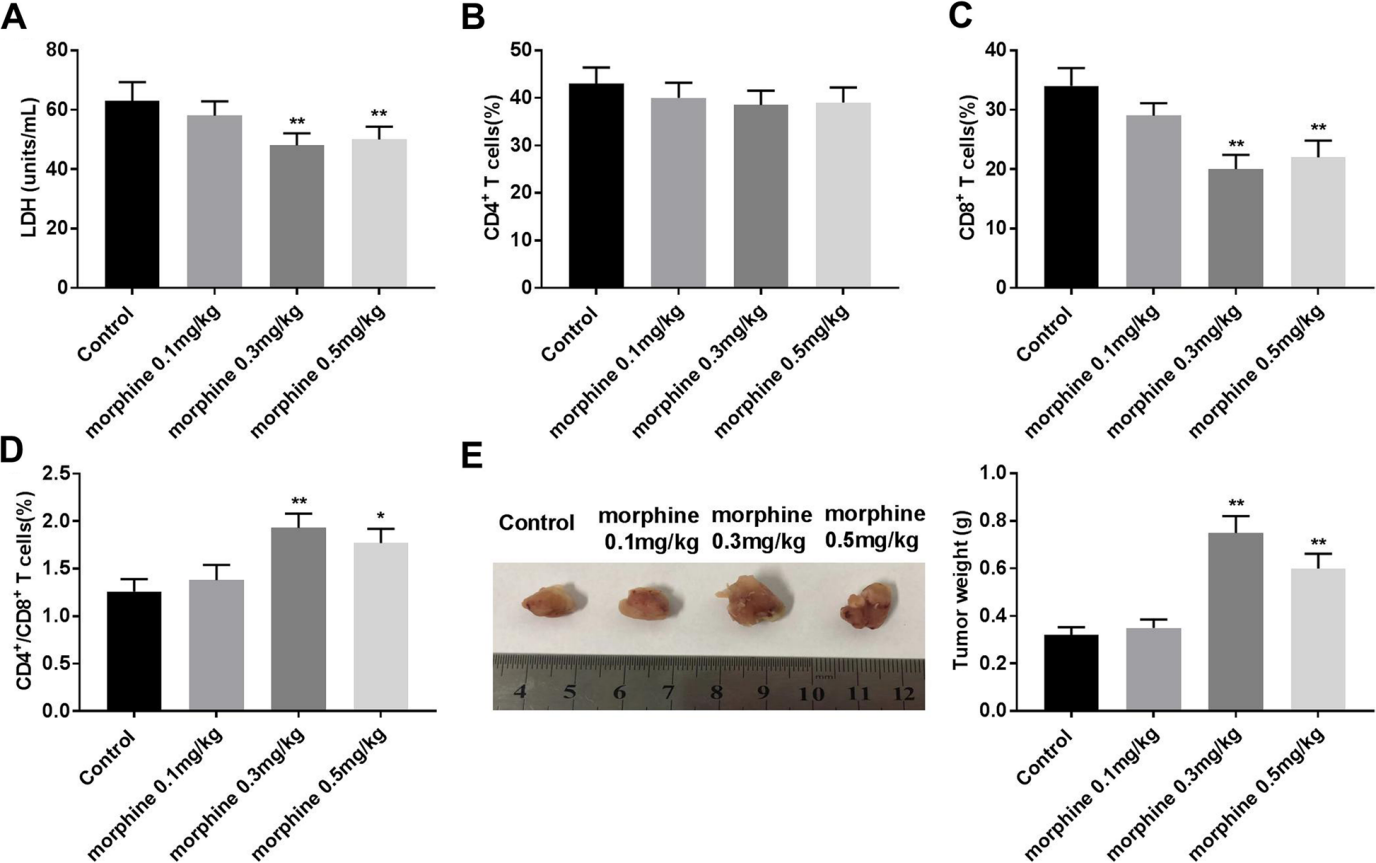
Morphine decreases LDH release and the percentage of CD8^+^ T cells, increased the ratio of CD4^+^/CD8^+^ T cells, and promoted the tumor weight. **A** The LDH release was detected using a LDH kit. **B**-**D** The numbers of CD4^+^ T and CD8^+^ T cells, and the ratio of CD4^+^/CD8^+^ T cells in control and morphine groups. **E** The picture of the tumor and weight of the tumor. *P < 0.05, **P < 0.01 versus control

LDH release assay was used to determine the cytotoxicity to A549 cells. The activation of CD4^+^ and CD8^+^ T cells is critical for an effective immune response [21, 29]. In addition, the ratio of CD4^+^/CD8^+^ T cells may be a critical parameter of anti-tumor immunity [29, 30]. Morphine treatment reduced the LDH level (Fig. 5A, *P* < 0.01), but did not distinctly change the number of CD4^+^ T cells in peripheral blood (Fig. 5B). The number of CD8^+^ T cells was reduced (Fig. 5C, *P* < 0.01), while the ratio of CD4^+^/CD8^+^ T cells was increased by morphine (0.3 mg/kg and 0.5 mg/kg) treatment (Fig. 5D, *P* < 0.05). In addition, morphine treatment (0.3 mg/kg and 0.5 mg/kg) promoted the weight of the tumor compared with control (Fig. 5E, *P* < 0.05). The data showed that morphine treatment significantly decreased cytotoxicity to A549 cells and facilitated tumor immune escape and tumor growth.

### MAEL silencing reverses the effects of morphine on immune cells

**Fig. 6:**
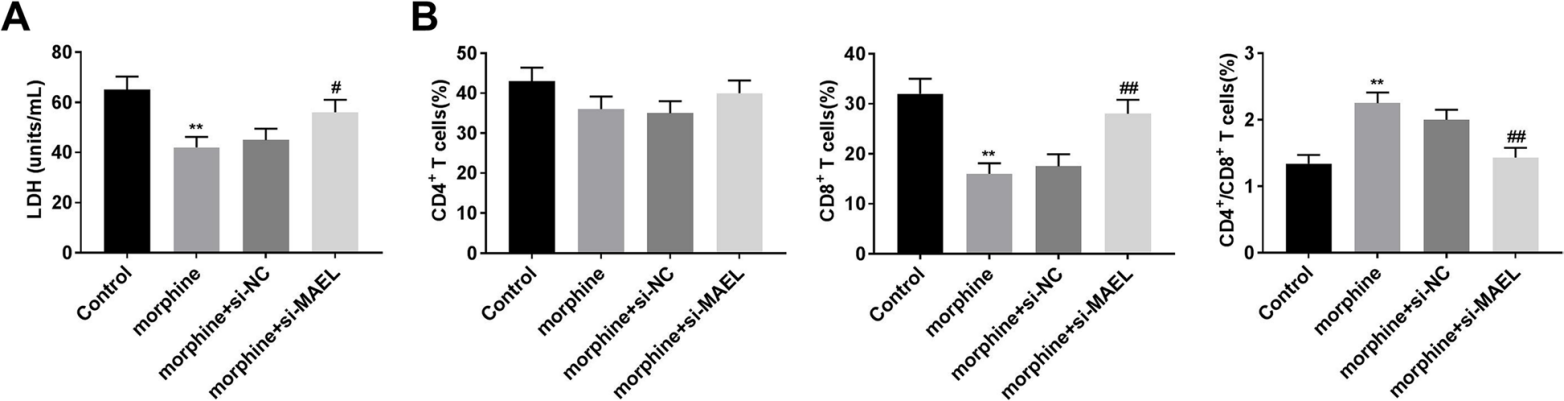
MAEL silencing reverses the effects of morphine on immune cells. **A** The LDH level was detected using a LDH kit. **B** The numbers of CD4^+^ T and CD8^+^ T cells, and the ratio of CD4^+^ /CD8^+^ T cells in control, morphine, morphine+si-NC, and morphine+si-MAEL groups. **P < 0.01 versus control. ^#^*P* < 0.05, ^##^*P* < 0.01 versus morphine+si-NC

MAEL silencing reversed the effect of morphine treatment on the LDH level (Fig. 6A, *P* < 0.05). The change of CD4+ T cells did not distinctly difference after MAEL silencing (Fig. 6B). Furthermore, MAEL silencing reversed the effects of morphine on the number of CD8^+^ T cells and the ratio of CD4^+^ T/CD8^+^ T cells (Fig. 6B, *P* < 0.01). The result showed that MAEL is involved in the regulation of morphine on the immune response.

## Discussion

Lung cancer is the third most common cancer. Cancer patients generally show immune suppression [31]. The host immune system, particularly cellular immunity, is closely associated with tumor occurrence, development, and metastasis of malignant tumors [32]. Studies have demonstrated that tumor immune evasion is one of the hallmarks of malignant tumors and represents an important step in tumor development [32]. In this research, we proved that morphine can regulate immune factors and immune cells, and morphine inhibits the immune response via up-regulating MAEL in lung cancer cells.

Morphine is a highly effective analgesic, which is widely used to relieve the pain and suffering of cancer patients [17]. Studies have suggested that morphine can promote the growth and spread of cancer, such as prostate cancer, breast cancer, and NSCLC [18]. Moreover, opioid alkaloids (morphine and heroin) have immunosuppressive effects in vivo and in vitro [33]. Morphine has analgesic activity and improvement of postoperative immunosuppression in patients undergoing abdominal surgery for uterine cancer [34]. Morphine-3-glucuronide up-regulates PD-L1 expression in NSCLC cells, and eventually promotes tumor immune escape [35]. Therefore, based on this, we speculated morphine can regulate the immune response in lung cancer. In this research, morphine up-regulated PD-L1 expression in A549 cells and H460 cells. Meanwhile, we also proved the effects of morphine on other immune factors, the expression of TGF-β and IL-10 were increased, while IL-2 expression was reduced. The result indicated that morphine induced tumor immune escape by regulating immune factors expression. Moreover, morphine decreases natural killer cell cytotoxicity and T cell subtypes (CD4^+^ and CD8^+^) content [36]. In this research, morphine decreased LDH level, the number of CD4^+^ T and CD8^+^ T cells, as well as the ratio of CD4^+^/CD8^+^ T cells was increased. Hence, the balance of CD4^+^ T and CD8^+^ T cells is pivotal for lung cancer prognosis, and morphine-induced tumor immune escape was further supported. Consequently, these results suggested that lung cancer is related to immune response, and morphine suppressed the immune system by regulating immune factors and immune cells in lung cancer.

Activation of Nrf2 can promote lung tumor development and associates with poor prognosis [37]. PTEN is considered a tumor suppressor. It regulates important cellular processes such as survival and proliferation [38]. Loss of PTEN is associated with poor clinical outcomes in NSCLC patients [39]. PTEN/PD-L1 axis regulates the tumor immune response in osteosarcoma [40]. Furthermore, the inhibition PTEN increases NRF2 protein levels [41]. In this study, we found that morphine-induced MAEL up-regulation can be reversed by PTEN in lung cancer cells. Based on these data, we suggested that morphine may regulate MAEL by regulation of the Nrf2/PTEN axis.

Some evidence has demonstrated the important role of MAEL in the progression of cancer. MAEL expression is associated with cell proliferation and invasion of colon cancer cells [42]. Knockdown of MAEL significantly inhibits cell proliferation in urothelial carcinoma of the bladder cell lines. MAEL is significantly highly expressed in breast cancer cells and related to some immune cells [43]. Similarly, in the present research, MAEL was up-regulated in A549 cells, and MAEL silencing inhibited cell viability and proliferation. Therefore, MAEL may exert an oncogenic role in the progression of lung cancer. Studies have indicated that tumor immune evasion is a critical process in the development of malignant tumors, and cancer germline genes may be involved in immune regulation in cancer [11]. Therefore, we speculated MAEL may promote immune evasion in lung cancer. In this study, MAEL silencing down-regulated the expression of PD-L1, TGF-β, and IL-10, and up-regulated IL-2 expression. Hence, the results suggested that MAEL may promote tumor immune evasion by regulating the expression of immune factors in lung cancer. Additionally, based on the immunosuppressive effects of morphine, we speculated morphine may exert effects by interacting with MALE in lung cancer. The result indicated that MAEL silencing reversed the effects of morphine on immune factors, CD8^+^ T cells, and the ratio of CD4^+^ T/CD8^+^ T cells. The results further demonstrated that MAEL silencing alleviated immune inhibition effects of morphine in lung cancer. Consequently, morphine may induce an immune-suppressive state by up-regulating MAEL expression in lung cancer.

In conclusion, this research demonstrated that morphine may suppress lung cancer immune by up-regulating the expression of MAEL. Furthermore, MAEL silencing may alleviate immune inhibition. This is worthy of further clinical study to validate in lung cancer patients.

## Supplementary Information

**Additional file 1.**

## Data Availability

The datasets generated during and/or analysed during the current study are available from the corresponding author on reasonable request.

## Abbreviations

LDH: Lactate dehydrogenase
MAEL: Maelstrom
PD-L1: Programmed death-ligand 1
TGF-β: Transforming growth factor beta
IL: Interleukin
NSCLC: Non-small cell lung cancer
SCLC: Small cell lung cancer
MSD: MAEL-specific domain
HMG: High mobility group
qRT-PCR: Quantitative real-time PCR
ELISA: Enzyme-linked immunosorbent assay
PBMCs: Peripheral blood mononuclear cells
Nrf2: Nuclear factor erythroid 2-related factor 2
PTEN: Phosphatase and tensin homologue
