# Supplementary material for: Morphine suppresses the immune function of lung cancer by up-regulating MAEL expression
Source: BMC Pharmacol Toxicol. 2022 Dec 7;23:92. doi: 10.1186/s40360-022-00632-z (PMC9730686; doi:10.1186/s40360-022-00632-z)

The expression of PD-L1, TGF-β, IL-2, and IL-10 was detected by western blot.

Figure1B GAPDH

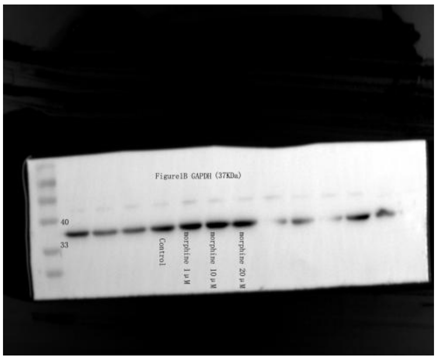


Figure1B IL-2

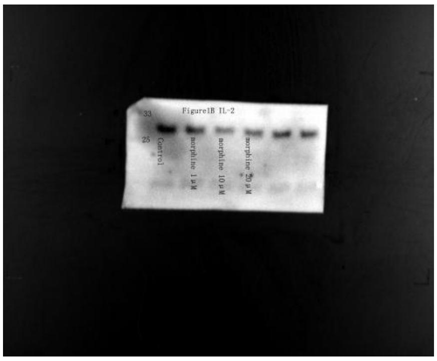


Figure1B IL-10

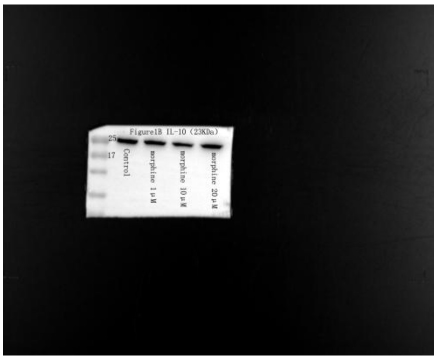

Figure1B PD-L1

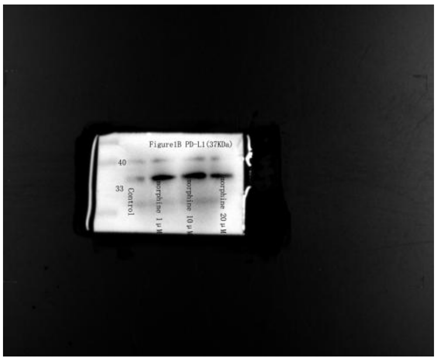


Figure1B TGF-β

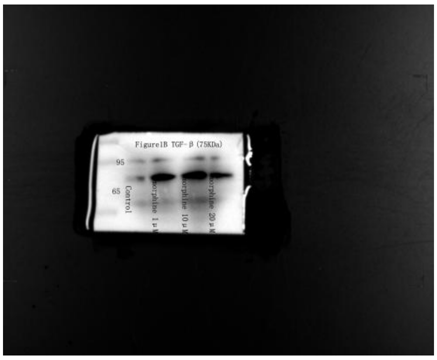

Supplement: Supplementary file 1 — Additional file 1. [file 40360_2022_632_MOESM1_ESM.zip › Western blot original images of Figure 1B.pdf]
