# Supplementary figures and images for: Morphine suppresses the immune function of lung cancer by up-regulating MAEL expression
Source: BMC Pharmacol Toxicol. 2022 Dec 7;23:92. doi: 10.1186/s40360-022-00632-z (PMC9730686; doi:10.1186/s40360-022-00632-z)

Figure2F GAPDH

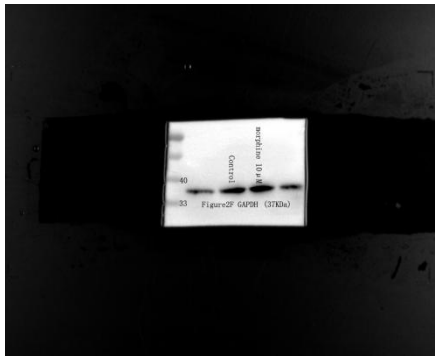

Figure2F MAEL

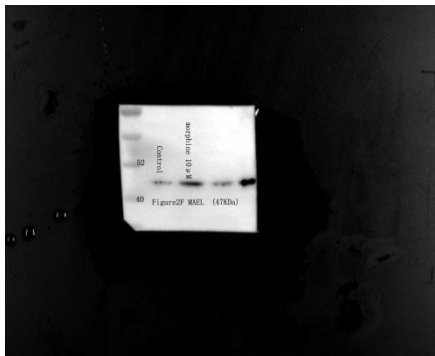

Supplement: Supplementary file 1 — Additional file 1. [file 40360_2022_632_MOESM1_ESM.zip › Western blot original images of Figure 2F.pdf]

Figure2H GAPDH

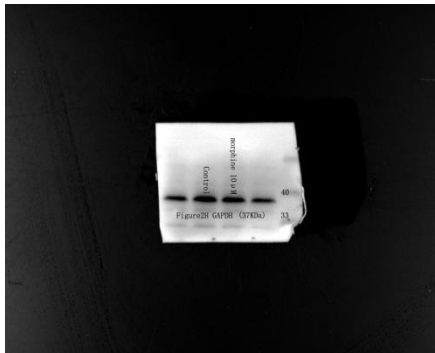

Figure2H MAEL

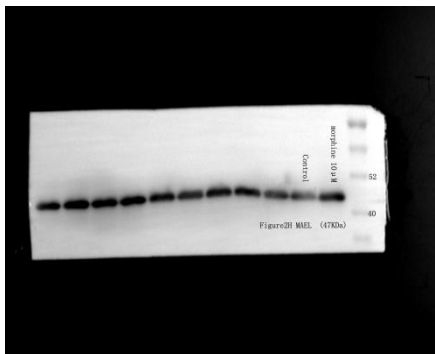

Supplement: Supplementary file 1 — Additional file 1. [file 40360_2022_632_MOESM1_ESM.zip › Western blot original images of Figure 2H.pdf]

Figure4G GAPDH

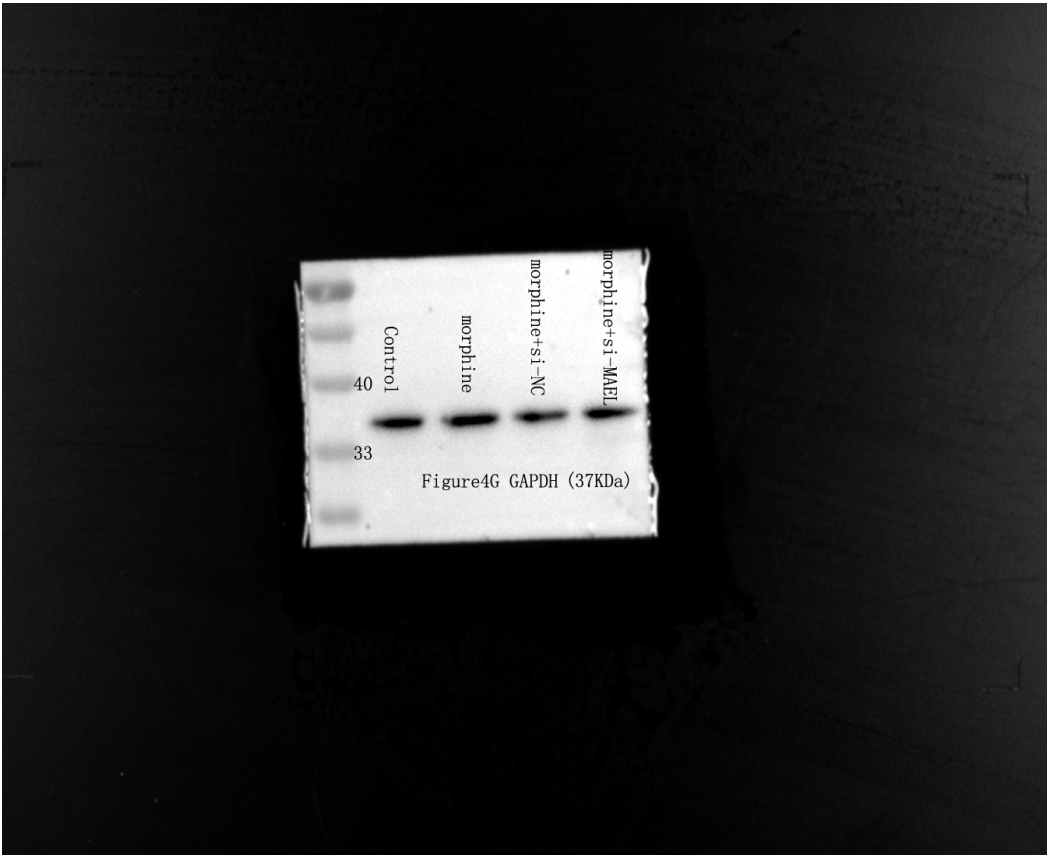

Figure4G PD-L1

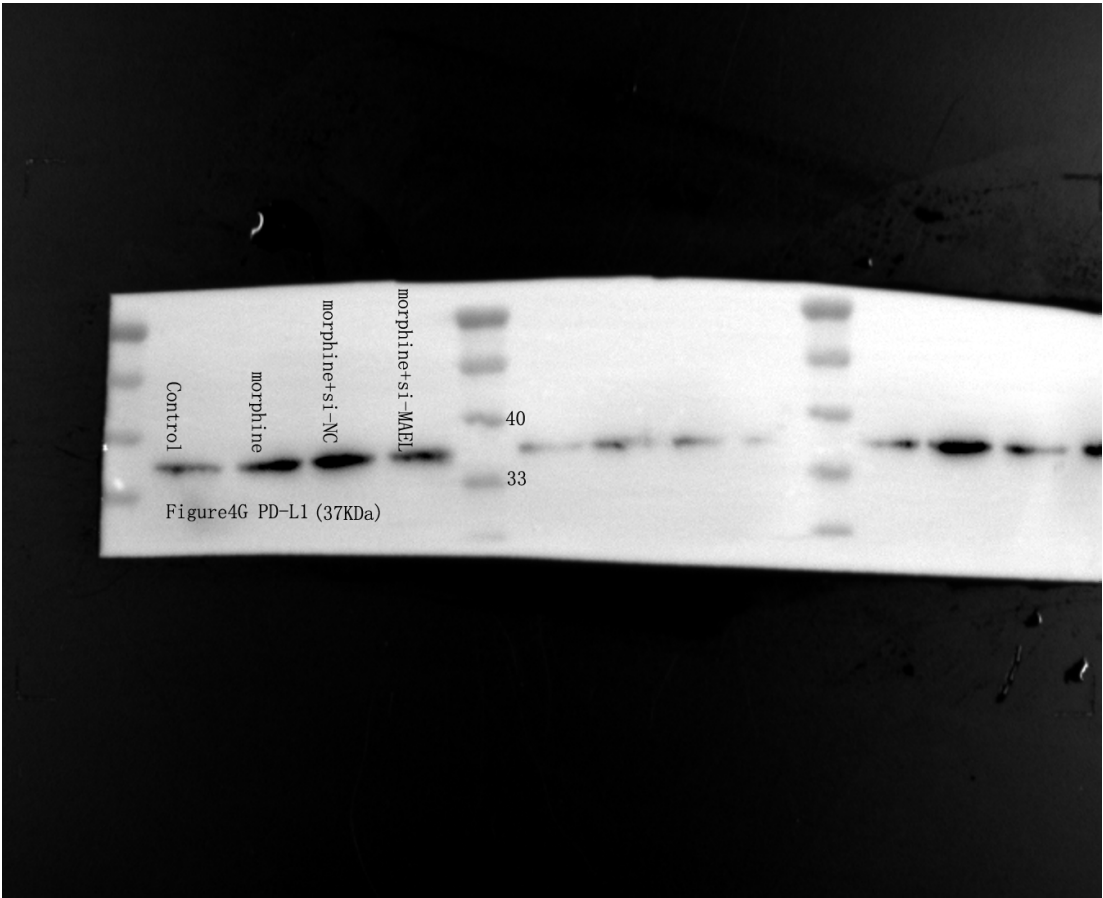

Figure4G TGF-β

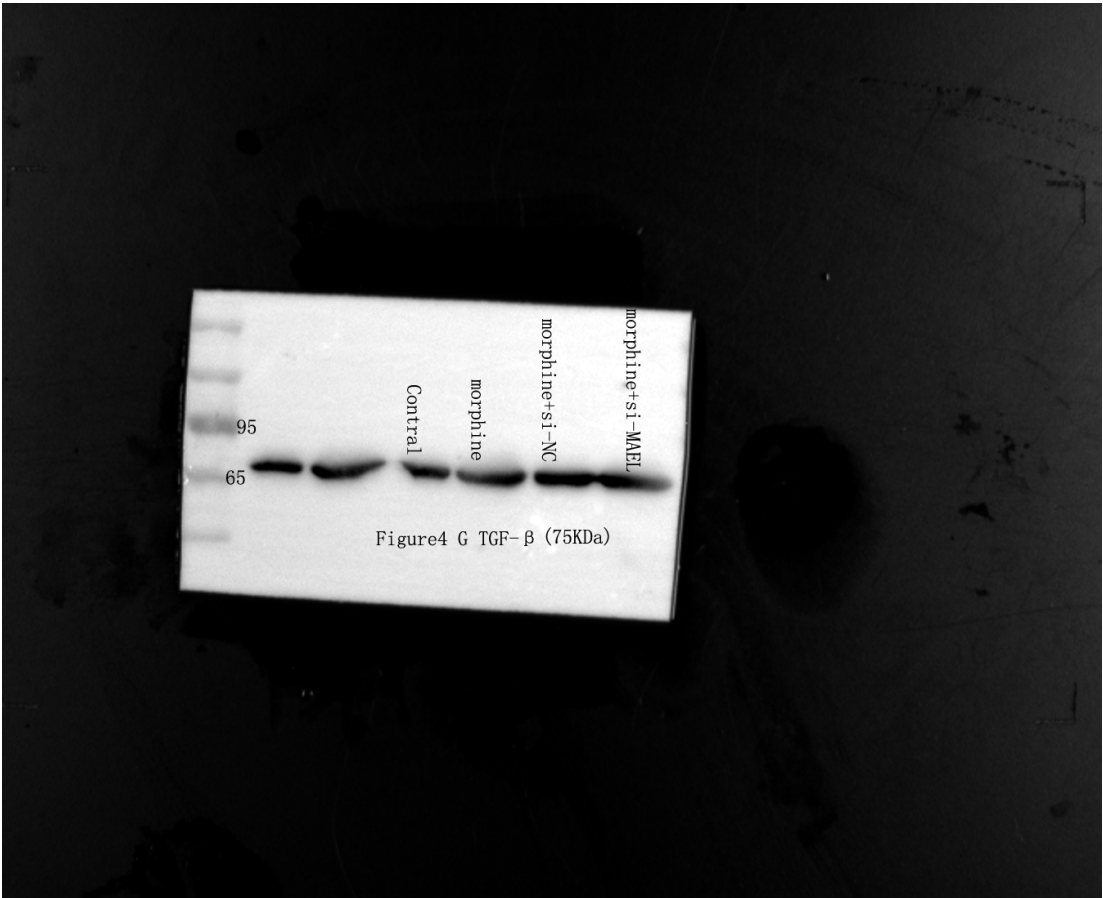

Supplement: Supplementary file 1 — Additional file 1. [file 40360_2022_632_MOESM1_ESM.zip › Western blot original images of Figure 4G.pdf]
